# DNA Sequence Variants and Protein Haplotypes of Casein Genes in German Black Pied Cattle (DSN)

**DOI:** 10.3389/fgene.2019.01129

**Published:** 2019-11-08

**Authors:** Saskia Meier, Paula Korkuć, Danny Arends, Gudrun A. Brockmann

**Affiliations:** Faculty of Life Sciences, Albrecht Daniel Thaer Institute for Agricultural and Horticultural Sciences, Animal Breeding Biology and Molecular Genetics, Humboldt University of Berlin, Berlin, Germany

**Keywords:** sequencing, 1000 Bull Genomes Project, bovine, SNP, comparative genomics, endangered

## Abstract

Casein proteins were repeatedly examined for protein polymorphisms and frequencies in diverse cattle breeds. The occurrence of casein variants in Holstein Friesian, the leading dairy breed worldwide, is well known. The frequencies of different casein variants in Holstein are likely affected by selection for high milk yield. Compared to Holstein, only little is known about casein variants and their frequencies in German Black Pied cattle (“Deutsches Schwarzbuntes Niederungsrind,” DSN). The DSN population was a main genetic contributor to the current high-yielding Holstein population. The goal of this study was to investigate casein (protein) variants and casein haplotypes in DSN based on the DNA sequence level and to compare these with data from Holstein and other breeds. In the investigated DSN population, we found no variation in the alpha-casein genes *CSN1S1* and *CSN1S2* and detected only the CSN1S1*B and CSN1S2*A protein variants. For *CSN2* and *CSN3* genes, non-synonymous single nucleotide polymorphisms leading to three different β and κ protein variants were found, respectively. For β-casein protein variants *A* *^1^*, *A* *^2^*, and *I* were detected, with CSN2*A *^1^* (82.7%) showing the highest frequency. For κ-casein protein variants *A*, *B*, and *E* were detected in DSN, with the highest frequency of CSN3*A (83.3%). Accordingly, the casein protein haplotype CSN1S1*B-CSN2*A *^1^*-CSN1S2*A-CSN3*A (order of genes on BTA6) is the most frequent haplotype in DSN cattle.

## Introduction

The German Black Pied cattle (DSN, “Deutsches Schwarzbuntes Niederungsrind”) is a dual-purpose breed for milk and beef production. DSN is considered the founder population of the high-yielding Holstein Friesian breed (Köppe-Forsthoff, 1967; Grothe, 1993). The DSN ancestors have their roots in the German and Dutch North Sea coast region. While DSN cattle produce about 2,500 kg less milk per lactation compared to German Holstein, they were almost entirely replaced by Holstein and DSN became an endangered breed with currently about 2,800 cows registered in Germany. Nevertheless, with 4.3% fat and 3.7% protein, milk from DSN cows contains more protein and fat compared to Holstein (RBB Rinderproduktion Berlin-Brandenburg GmbH, 2016). Moreover, DSN cattle are considered to be more robust and fertile.

To preserve the DSN breed and conserve the genetic diversity, farmers are financially compensated for the lower milk yield by the EU and the German government. The close genetic relationship to Holstein makes a genetic comparison between the original DSN and Holstein interesting with respect to differences in milk yield and protein composition.

Genes known to influence protein content and composition in milk are the casein genes *CSN1S1*, *CSN2*, *CSN1S2*, and *CSN3*, encoding the casein proteins alpha S1 (α_S1_), beta (β), alpha S2 (α_S2_), and kappa (κ), respectively (Ferretti et al., 1990; Threadgill and Womack, 1990), which are located in the given order on BTA6 in the so-called casein gene cluster, which spans ∼250 kb (Boettcher et al., 2004). All caseins account for about 75% of the milk protein content (Gallinat et al., 2013); the remaining 25% are whey proteins. Several single nucleotide polymorphisms (SNPs) and insertions or deletions in exons of these casein genes are known to change their protein sequences, resulting in different casein variants. In the *Bos* genus, 10 protein variants for α_S1_- (*A*, *B*, *C*, *D*, *E*, *F*, *G*, *H*, *I*, and *J*), 15 for β- (*A* *^1^*, *A* *^2^*, *A* *^3^*, *B*, *C*, *D*, *E*, *F*, *G*, *H* *^1^*, *H* *^2^*, *I*, *J*, *K*, and *L*), 5 for α_S2_- (*A*, *B*, *C*, *D*, and *E*), and 11 for κ-casein (*A*, *B*, *C*, *E*, *F* *^1^*, *F* *^2^*, *G* *^1^*, *G* *^2^*, *H*, *I*, and *J*) have been reported (**Table 1**). Additional variants in the upstream gene regions could affect the expression of the casein genes and influence the amount and ratio of different caseins in the milk (Martin et al., 2002). Casein polymorphisms were found to affect milk processing and cheese making properties as well as the digestibility in human nutrition, hypoallergenic reactivity, and the risk of cardiovascular diseases and diabetes, for example (Caroli et al., 2009).

**Table 1:** Known protein variants

Gene	Protein	Variants
*CSN1S1*	α_S1_	*A*, *B*, *C*, *D*, *E*, *F*, *G*, *H*, *I*, *J*
*CSN2*	β	*A* *^1^*, *A* *^2^*, *A* *^3^*, *B*, *C*, *D*, *E*, *F*, *G*, *H* *^1^*, *H* *^2^*, *I*, *J*, *K*, *L*
*CSN1S2*	α_S2_	*A*, *B*, *C*, *D*, *E*
*CSN3*	κ	*A*, *B*, *C*, *E*, *F* *^1^*, *F* *^2^*, *G* *^1^*, *G* *^2^*, *H*, *I*, *J*

In this table, we list all known variants for the casein genes published in recent literature for the CSN1S1, CSN2, CSN1S2, and CSN3 genes and their corresponding proteins in Bos genus (Ibeagha-Awemu et al., 2007; Caroli et al., 2010; Gallinat et al., 2013).

While many studies investigated the casein gene cluster in Holstein and other breeds (Ng-Kwai-Hang et al., 1984; Velmala et al., 1995; Formaggioni et al., 1999; Boettcher et al., 2004; Gallinat et al., 2013), so far only little is known about the genetic diversity of the casein cluster in DSN cattle. In a former study of β- and κ-casein variants in DSN cattle, homozygous carriers of the β-casein variant *A* *^2^* showed a tendency for higher milk, fat, and protein yield with lower fat and protein percentages, while κ-casein variants tended to have an influence on the protein percentage (Freyer et al., 1999). Since DSN has not been selected for protein variants in the recent past, but for other important traits such as milk yield and udder conformation, an indirect selection for specific casein variants could have happened as a by-product. Because of the close proximity of the four casein genes in the bovine genome, the casein genes are not inherited independently, but are often transmitted from parents to offspring as a single haplotype. Therefore, it is very useful to determine the frequency not only for single protein variants but also for each “comprehensive haplotype” made by building a haplotype out of protein variants found in the four casein genes using the sequential order in which these genes are found in the casein cluster. Such haplotypes for the casein gene cluster were described for many dairy breeds using sequence variation within coding regions (Ikonen et al., 2001; Caroli et al., 2003; Boettcher et al., 2004), in promoter regions (Jann et al., 2004; Ahmed et al., 2017) or microsatellites (Velmala et al., 1995). Some studies provided evidence for a correlation between casein haplotypes and milk yield, fat, and protein percentage (Velmala et al., 1995; Braunschweig et al., 2000; Ikonen et al., 2001; Boettcher et al., 2004; Braunschweig, 2008; Nilsen et al., 2009).

In the DSN cattle, the frequencies of single casein protein variants and casein protein haplotypes recently have been investigated by isoelectric focusing of milk samples (*N* = 1,219) (Hohmann et al., 2018). In British Friesian, a breed that has similar ancestors and a similar breeding history like DSN, casein haplotypes were examined on the basis of genotype data (*N* = 51) (Jann et al., 2004).

In the current study, we used whole-genome sequencing data of the DSN population and additional data from the 1000 Bull Genomes Project (Daetwyler et al., 2014; http://www.1000bullgenomes.com/) to examine and compare the sequence of all casein genes including the 1-kb upstream regulatory region. Our aim is to compare the DSN population with 13 other cattle breeds. This comparison is undertaken to investigate the genetic diversity of missense variants in the casein gene cluster across these cattle breeds and might provide selectable casein variants and/or haplotypes to improve DSN breeding.

## Material and Methods

### Sequencing Data

In order to characterize DSN casein sequence variants, the raw sequence variants of *Bos taurus* animals available from the 1000 Bull Genomes Project Run 6.0 were used (http://www.1000bullgenomes.com/; Daetwyler et al., 2014). Animals that shared high genetic similarity (>0.99 relative Manhattan distance; Korkuć et al., 2019), which could not be explained by kinship, were removed from the dataset. Furthermore, only breeds with at least 30 animals were selected for the analyses, so that the final dataset contained 14 different *B. taurus* breeds (30 DSN, 541 Holstein Friesian, 276 Angus, 217 Simmental, 148 Brown Swiss, 127 Charolais, 82 Limousin, 75 Hereford, 66 Jersey, 56 Danish Red, 54 Montbéliarde, 53 Fleckvieh, 52 Gelbvieh, and 44 Normande).

Filtering of raw SNP data was performed as described in Daetwyler et al. (2014), except we did not apply the proximity filter, which keeps only the highest quality SNP within 3 bp to increase the number of investigated SNPs in the casein cluster. In addition, we required at least three reads mapped to the reference and/or alternative allele to be considered a trustworthy SNP call; otherwise, the SNP genotype for that animal was set to missing. Only variants were investigated which are polymorphic in at least one breed.

The 30 DSN cattle in the 1000 Bull Genomes dataset were selected to best represent the current DSN population. The DSN population submitted includes 13 cows (mostly bull mothers) and 17 artificial insemination bulls. Due to the small population size, relationships between DSN cattle exist. Animal selection criteria for the other breeds from the 1000 Bull Genomes Project are not known.

### Investigated DNA Sequence Region

Genomic positions, reference genome, and protein sequences of the casein genes were obtained from Ensembl Release 93 (Zerbino et al., 2018) based on UMD3.1 assembly (Zimin et al., 2009). Sequence variants located within the casein genes *CSN1S1*, *CSN1S2*, *CSN2*, and *CSN3* (**Supplementary Table 1**) and 1,000 bp upstream were selected for analyses. The sequence variants were examined and categorized into variant types based on their genomic locations (1,000 bp upstream, 5′-UTR, intron, synonymous, missense, splice region, 3′-UTR) using the Ensembl Variant Effect Predictor (McLaren et al., 2016).

The lowest detectable allele frequency in DSN was 1/60 (0.017) as the minimum number of animals per breed was set to 30. So an allele frequency of 0.017 implies a single heterozygous animal within the population.

A comparison of the SNP annotation of the genes in the casein cluster to the rest of the genomic SNP was performed using all SNP variants annotated by the 1000 Bull Genomes Project (Hayes and Daetwyler, 2019). However, while our analysis of the casein cluster does not include intergenic variants, we recalculated the annotation percentages in the 1000 Bull dataset after removing the “intergenic variant” category. A comparison between the casein cluster and the rest of the genome can be found in **Supplementary Table 7**.

Haplotypes and haplotype frequency of protein-coding variants were estimated if at least two protein-coding variants were present. Haplotype analysis was performed using the function haplo.group from R package haplo.stats with the default settings (Sinnwell and Schaid, 2018). In order to assess the similarity of cattle breeds with regard to their haplotypes, Euclidean distances of protein variants and haplotype frequencies between all breeds were calculated. The resulting distance matrix was used to cluster (using average linkage) the cattle breeds hierarchically and to generate a dendrogram with standard R plot routines. All other plots were generated using the R package ggplot2 (Wickham et al., 2016).

Protein variants with a minimum frequency of 5% in a single breed were used to build comprehensive haplotypes across all four casein genes. Haplotypes are named according to the ordered position of the casein genes on the chromosome (*CSN1S1-CSN2-CSN1S2-CSN3*) and the variant name of each individual casein protein, e.g., *B-A* *^1^* *-A-A* for CSN1S1*B–CSN2*A *^1^* –CSN1S2*A–CSN3*A. This way of coding casein variants was proposed by Caroli et al.; more information about casein (haplotype) coding can be found in their 2009 paper (Caroli et al., 2009).

## Results

### Distribution of DNA Sequence Variants in Casein Genes and Upstream Regions

In total, 892 SNPs were detected within the four casein genes (*CSN1S1*, *CSN2*, *CSN1S2*, and *CSN3*) and their 1,000-bp upstream regions. Most of the detected variants were intron variants (87.3%), followed by variants in the 1,000-bp upstream gene region (5.8%), and missense variants (2.2%). Remaining SNPs were synonymous variants (1.2%), located in the 3′-UTR (2.2%), splice region (0.7%), or in the 5′-UTR region (0.4%) (**Table 2** and **Supplementary Table 2**).

**Table 2:** SNP density.

Gene	Upstream	Intron	Exon	Missense	Synonymous
*CSN1S1*	22.0	17.3	3.4	1.7	1.7
*CSN2*	10.0	15.6	8.7	6.1	2.6
*CSN1S2*	8.0	9.6	5.8	2.5	3.3
*CSN3*	12.0	17.4	9.5	8.3	1.2
Total	13.0	14.6	6.2	4.0	2.2

SNP density per 10 kb in the upstream (+1,000 bp), intron and exon (split into missense and synonymous variants) regions of the casein genes CSN1S1, CSN2, CSN1S2, and CSN3.

Comparison of casein SNPs to the 1000 Bull Genomes whole-genome SNP dataset showed that the percentages detected in the casein cluster are similar to the whole-genome annotation frequencies (intron variants 84.7%, upstream region 11.4%, missense variants 1.4%, synonymous variants 1.4%, 3′-UTR 0.7%, splice region 0.2%, and 5′-UTR 0.2%) (**Supplementary Figure 1** and **Supplementary Table 7**).

SNP density was calculated for the average number of SNPs per 10 kb for upstream (+1,000 bp), intron and exon regions of the four casein genes (**Table 2**). The highest SNP density over all four genes was found in the introns (14.57 SNPs per 10 kb), followed by upstream gene regions (13.00 SNPs per 10 kb) and exons (6.22 SNPs per 10 kb). *CSN3* had the highest density of intronic DNA variants (17.44 SNPs per 10 kb) and exon regions (9.46 SNPs per 10 kb), while *CSN1S1* had the lowest SNP density in the exons (3.36), but the highest in the upstream region (22.00).

In DSN, 254 of 892 sequence variants over all four casein genes were detected (**Supplementary Table 3**). Six SNPs were found to be novel. This means that these SNPs were not found in the dbSNP and/or EVA database; this was investigated using the Ensemble genome browser (Release 93) which integrates both these databases. One in intron 6 of *CSN1S1* (BTA6:87147250 G/A) found in DSN and Holstein. One in intron 14 within the splice region of *CSN1S1* (BTA6:87155332 C/T) found in DSN, Holstein, and Fleckvieh. Another novel SNP that was found in intron 2 of *CSN3* (BTA6:87382140 T/C) was segregating in most of the investigated breeds. The alternative allele frequency (AAF) of this SNP is similar in DSN and Danish Red (AAF_(DSN)_ = 28.3%, AAF_(Danish_ _Red)_ = 21.7%), while all other breeds showed an alternative allele frequency <10%. Interestingly, in *CSN2*, three novel SNPs were found in a single DSN bull only, one of them in intron 1 (BTA6:87186177 G/A) and two in intron 4 (BTA6:87185025 T/A and BTA6:87184912 C/G).

**Figure 1:**
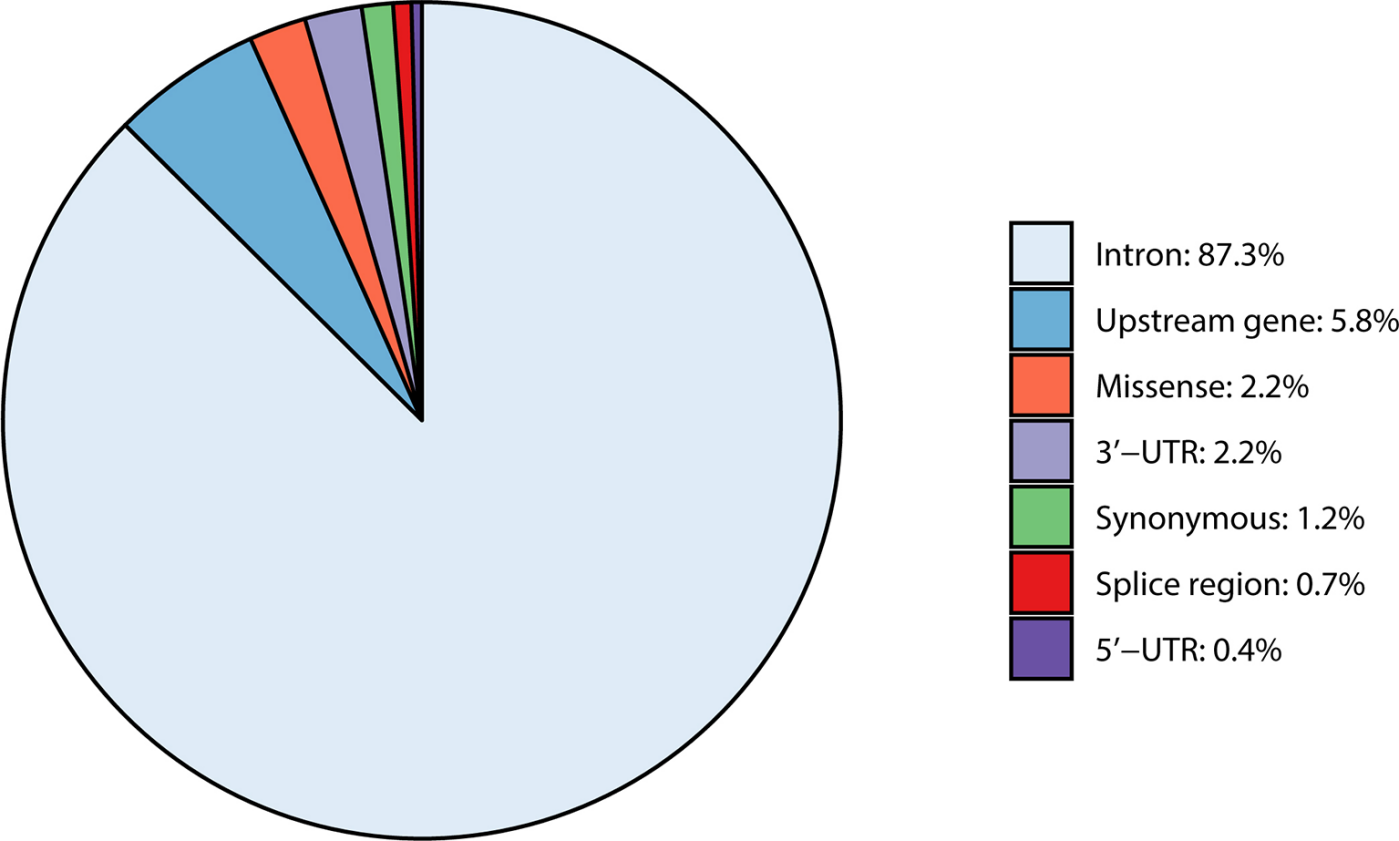
Overview of variant types occurring within the four casein genes *CSN1S1*, *CSN1S2*, *CSN2*, and *CSN3* including their 1,000-bp upstream region.

**Figure 2:**
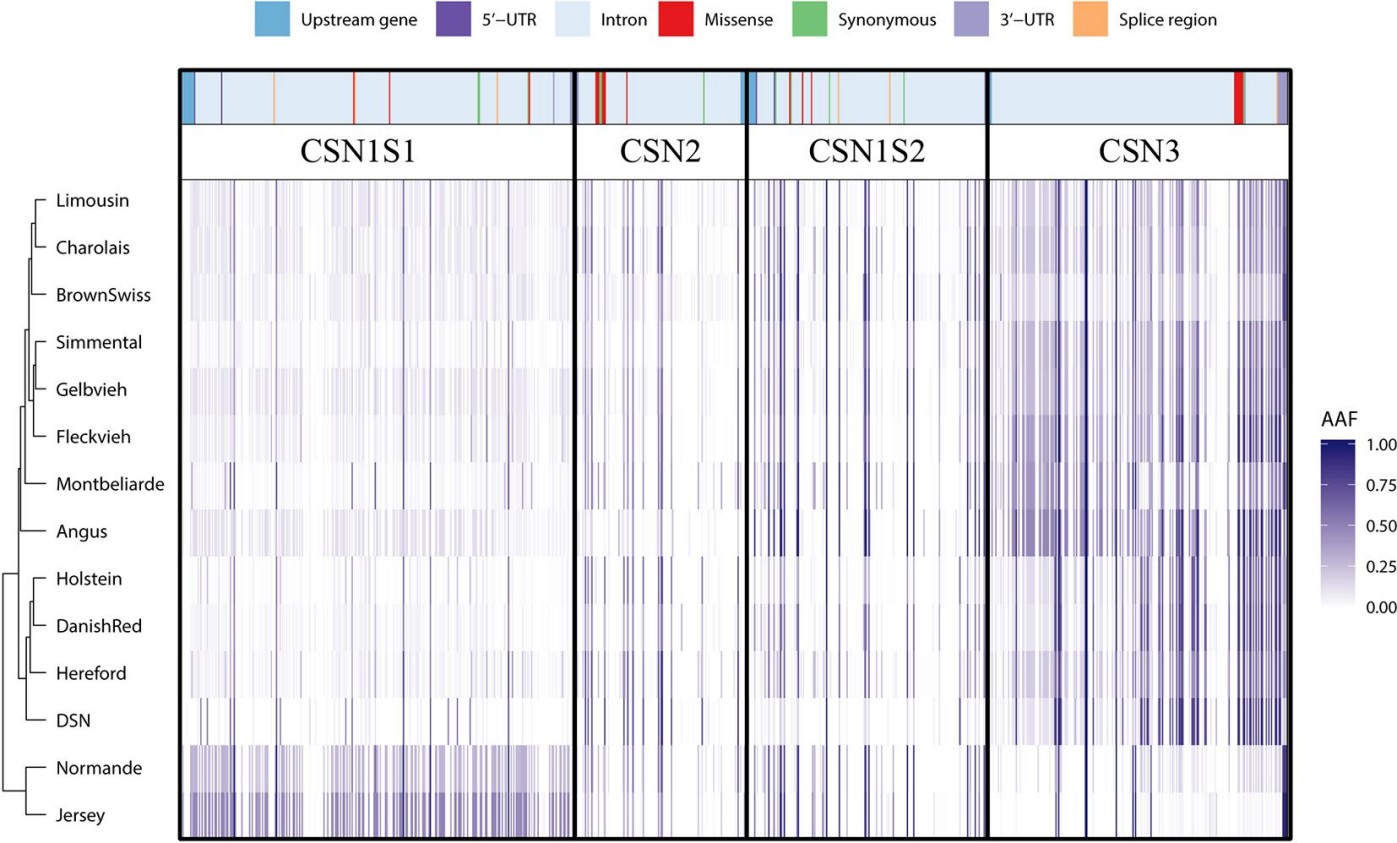
Clustering of per-breed alternative allele frequency for the detected sequence variants in the casein genes *CSN1S1*, *CSN2*, *CSN1S2*, and *CSN3* including their 1,000-bp upstream region. The respective variant types are presented above the alternative allele frequencies. It should be noted that the clustering is mainly based on intron variants (*light blue areas*) as they make up 87.3% of all detected variants.

The alternative allele frequency of all SNPs in the four casein genes differs between the investigated breeds. Through clustering of the 892 SNPs based on the respective alternative allele frequency per breed, distinct relationships between the breeds can be observed (**Figures 1** and **2**). The alternative allele frequencies of the sequence variants across all casein genes showed breed-specific differences. Overall, the alternative allele frequencies of DSN are most similar to those of Danish Red (dual-purpose breed), Holstein (milk production breed), and Hereford (beef production breed). DSN show very low alternative allele frequency for SNPs in *CSN1S1* and *CSN1S2*, but higher ones for SNPs in *CSN2* and *CSN3*. In contrast to all other breeds, Normande and Jersey had high and low alternative allele frequency in *CSN1S1* and *CSN3*, respectively. As such, these two breeds also cluster together on the lower side of the dendrogram (**Figure 2**). The relationship between all investigated breeds based on all genome-wide SNPs in the 1000 Bull Genomes Project showed a close relatedness between DSN, Holstein, and Danish Red (**Supplementary Figure 2**).

### Casein Protein Variants

#### CSN1S1

Protein variants CSN1S1*B and CSN1S1*C were detected in at least one breed. In DSN, only the CSN1S1*B variant was detected (**Table 3**). Variants CSN1S1*A and CSN1S1*C were not observed among the 30 sequenced DSN animals. In Gelbvieh, Holstein, and Danish Red, the frequency of the CSN1S1*C variant was also low (<1%). In contrast, Limousin, Brown Swiss, and Fleckvieh had higher frequencies of the CSN1S1*C variant (>10%). The highest protein variant frequency of the CSN1S2*C variant was detected in the Jersey (44.8%) and Normande (25.6%) breeds (**Supplementary Table 4**).

**Table 3:** Allele frequency of missense variants.

Variant of casein gene	BTA position^a^	Allele	Amino acid	Protein seq. position^b^	SNP ID	Variant frequency		
						DSN	HF	All breeds
CSN1S1*B	6:87157262	**A**/G	**Glu**/Gly	207 (192)	rs43703010	1.0	0.995	0.944
CSN2*A *^1^*	6:87181619	**T**/G	**His**/Pro	82 (67)	rs43703011	0.827	0.340	0.295
CSN2*A *^2^*	6:87181619	T/**G**	His/**Pro**	82 (67)	rs43703011	0.156	0.562	0.592
CSN2*I	6:87181542	T/**G**	Met**/Leu**	108 (93)	rs109299401	0.017	0.059	0.036
CSN1S2*A	6:87266177	**C**/T	**Ser**/Phe	23 (8)	rs441966828	1.0	1.0	0.994
CSN3*A	6:87390576	T/**C**	Ile/**Thr**	157 (136)	rs43703015			
	6:87390612	**C**/A	**Ala**/Asp	169 (148)	rs43703016	0.833	0.752	0.628
	6:87390632	**A**/G	**Ser**/Gly	176 (155)	rs43703017			
CSN3*B	6:87390576	**T**/C	**Ile**/Thr	157 (136)	rs43703015	0.133	0.203	0.341
	6:87390612	C/**A**	Ala/**Asp**	169 (148)	rs43703016			
CSN3*E	6:87390632	A/**G**	Ser/**Gly**	176 (155)	rs43703017	0.034	0.045	0.030

Allele frequencies of missense variants in CSN1S1, CSN2, CSN1S2, and CSN3 in DSN compared to Holstein Friesian (HF) and other breeds. For each variant, we list the alleles as ref/alt. In a bold font we highlight the SNP allele and resulting amino acid which causes the casein variant. As an example, the CSN2*A^1^ and CSN2*A^2^ variants are caused by a SNP on the same position 6:87181619. In the case of A^1^, the T-allele causes a histidine to be incorporated into the protein sequence. The A^2^ variant is defined as a G on the same position, leading to a proline in the resulting protein.

a Bos taurus autosome (BTA) CSN1S1*B (ENSBTAG00000007695), CSN2*A^2^ (ENSBTAG00000002632), CSN1S2*A (ENSBTAG00000005005), and CSN3*A (ENSBTAG00000039787).

b Positions of amino acids according to the reference protein sequence from Ensembl Release 93 UMD3.1 assembly. Positions in the mature protein are given in parentheses.

#### CSN2

Seven missense variants were found in the *CSN2* gene, of which five β-casein protein variants (*A* *^1^*, *A* *^2^*, *B*, *I*, and *F*) have a frequency of at least 5% in one breed. The distribution of those five most common β-casein protein variants differed in DSN compared to the other breeds. In DSN, the *A* *^1^* is the most common protein variant with a frequency of 82.7% compared to 30.0% in Holstein. The protein variants *A* *^2^* (15%) and *I* (2%) were found in DSN as well (**Table 3**). Variant *I* has not been described before for DSN (Jann et al., 2002; Caroli et al., 2009). The variants *B* and *F* were not detected in the examined DSN population, but were found in other breeds. Nine out of 14 breeds have a frequency of the *A* *^2^* variant of more than 50%, with the highest frequency in Angus (94.7%) (**Supplementary Table 5**).

#### CSN1S2

In the *CSN1S2* gene, three missense variants were found which correspond to protein variants CSN1S2*A, CSN1S2*C, and CSN1S2*D. In DSN only variant *A* was detected (**Table 3**), similar to Jersey, Montbéliarde, Normande, Fleckvieh, and Hereford. Additionally, in Holstein, CSN1S2*D was found with low frequencies (0.3%). Gelbvieh has the highest frequency for variant *D,* with 12.2%. The highest frequency of the *C* variant was found in Angus, with a frequency of 7.5% (**Supplementary Table 4**).

#### CSN3

Seven missense variants were found in the *CSN3* gene. The κ-casein variants *A*, *B*, and *E* have a frequency of at least 5% in one breed. In DSN, variant *A* is the most frequent (83.3%), followed by *B* (13.3%) and *E* (3.4%) (**Table 3**). CSN3*A is the most frequently detected variant in 10 out of the 14 breeds investigated. The highest frequency for the *B* variant was found in Jersey (96.0%), Brown Swiss (67.4%), Normande (84.6%), and Charolais (51.0%). The distribution of the *CSN3* protein variants in DSN are similar to Fleckvieh (CSN3*A = 84.4%, CSN3*B = 14.5%), although the *E* variant was not detected in Fleckvieh (**Supplementary Table 6**).

### Protein Haplotype Analysis Across the Casein Cluster

Across all casein genes, frequency of variants varied between the investigated breeds. Therefore, we performed a haplotype analysis across all protein variants of the four casein genes to position DSN relative to the other breeds.

Altogether, 37 haplotypes were constructed across all cattle breeds; 13 out of 37 haplotypes had a frequency higher than 5% in at least one breed. Out of the 13 haplotypes which met our inclusion criteria, five haplotypes showed a frequency >5%. For DSN, nine haplotypes could occur theoretically based on the number of casein protein variants across the casein cluster. Out of the expected haplotypes, seven were found. The most common haplotype in DSN was *B-A* *^1^* *-A-A* with a frequency of 71.1%. In contrast to DSN, the most frequent haplotype in Holstein (53.1%) as well as in seven other breeds was *B-A* *^2^* *-A-A* (**Table 4**).

**Table 4:** Haplotype frequencies for the casein cluster *CSN1S1-CSN2-CSN1S2-CSN3* for all breeds.

Haplotype	Total	Limousin	Angus	Hereford	Charolais	Simmental	Fleckvieh	Normande	Montbéliarde	Brown Swiss	Gelbvieh	Jersey	Danish Red	Holstein	DSN
N animals	1821	82	276	75	127	217	53	44	54	148	52	66	56	541	30
*B-A* *^2^* *-A-A*	0.424	0.419	0.644	0.359	0.331	0.446	0.415	0.100	0.072	0.115	0.490	0.045	0.136	0.531	0.116
*B-A* *^1^* *-A-A*	0.147	0.071	0.032	0.313	0.041	0.145	0.270	0.046	0.055	0.078	0.091		0.439	0.196	0.717
*B-A* *^2^* *-A-B*	0.141	0.196	0.075	0.026	0.286	0.178	0.147	0.081	0.304	0.500	0.133	0.047	0.132	0.056	0.024
*B-A* *^1^* *-A-B*	0.057	0.105	0.006	0.028	0.163	0.081		0.062	0.014	0.020	0.114	0.124	0.031	0.063	0.093
*B-B-A-B*	0.051	0.038	0.002	0.238	0.013	0.009		0.221		0.079		0.226	0.017	0.027	
*C-A* *^2^* *-A-B*	0.048	0.094	0.026	0.007	0.033	0.032		0.284		0.069		0.506	0.002	0.001	
*B-I-A-B*	0.035	0.029	0.007	0.007		0.020		0.162	0.151			0.052		0.061	0.017
*B-A* *^2^* *-A-E*	0.020		0.105			0.003							0.028	0.002	0.013
*C-A* *^2^* *-A-A*	0.019	0.018	0.020	0.023	0.028	0.049	0.117	0.020	0.034	0.051				0.002	
*B-B-A-A*	0.017				0.065	0.030	0.022		0.359	0.083				0.001	
*B-A* *^2^* *-D-B*	0.004										0.132		0.054		
*B-F-A-A*	0.005												0.136	0.003	
*B-A* *^2^* *-C-A*	0.011		0.075												
*Residual*	0.021	0.030	0.007		0.040	0.006	0.030	0.023	0.011	0.005	0.040		0.022	0.057	0.021

Haplotypes with at least 5% in one breed are shown.

**Figure 3:**
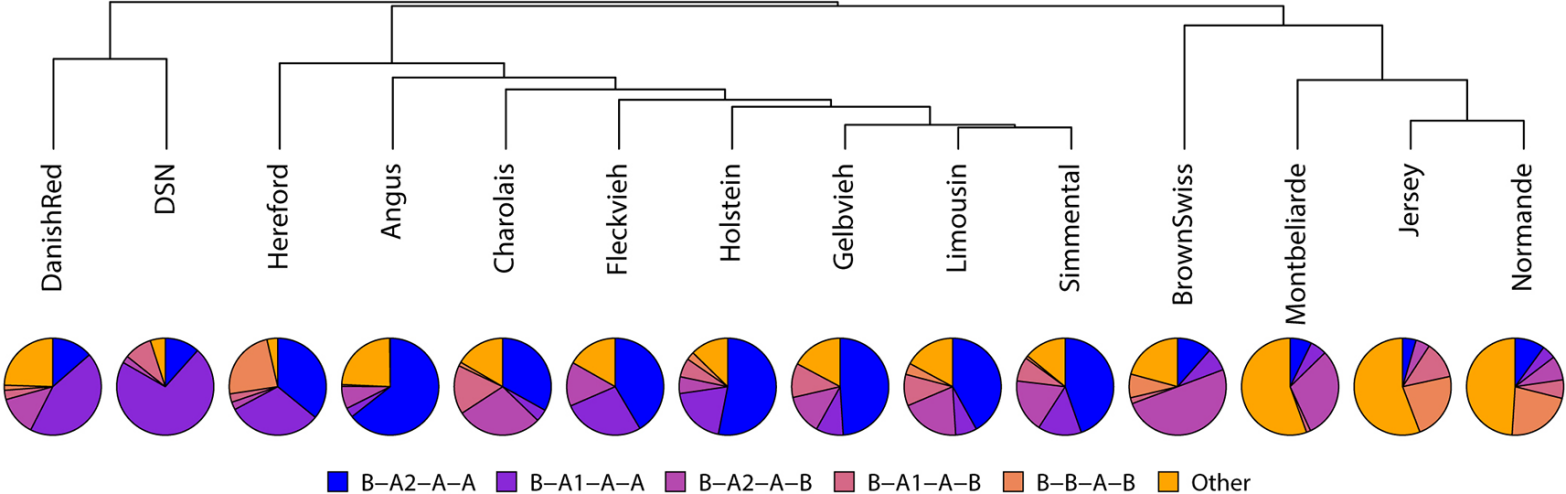
Haplotype analysis across the casein proteins *CSN1S1-CSN2-CSN1S2-CSN3* for the five most common haplotypes listed using the respective protein variant names. Haplotypes with a total frequency less than 5% are summarized as “Other.”

Because of their similarity in their comprehensive haplotype distribution, DSN and Danish Red cattle clustered closely together (**Figure 3**). Both show the highest frequency for the *B-A* *^1^* *-A-A* haplotype. Holstein clusters together with Hereford, Angus, Charolais, Fleckvieh, Gelbvieh, Limousin, and Simmental, which all show the highest frequency for the *B-A* *^2^* *-A-A* haplotype. The breeds Brown Swiss (*B-A* *^2^* *-A-B* = 50.0%), Montebéliarde (*B-A* *^2^* *-D-B* = 35.9%), Jersey (*C-A* *^2^* *-A-B* = 50.6%), and Normande (*C-A* *^2^* *-A-B* = 28.4%) cluster together, showing the highest proportion of other haplotypes.

## Discussion

### DNA Sequence Variants and New Alleles

Over the whole cattle genome, 0.6% of base pairs were polymorphic sequence variants in all breeds within the 1000 Bull Genomes Project (Sanchez et al., 2017). Within the casein cluster, we detected 0.4% of polymorphic sequence variants, which is an adequate result under consideration of the short region of about 250 kb on the bovine genome.

In the investigated casein region, intron variants are slightly more frequent with 87.3% in our study than in the whole cattle genome with an average of 84.7% (Hayes and Daetwyler, 2019). Upstream gene variants make up 11.4% of all SNPs in the whole cattle genome. In this study (1,000 bp upstream), only 5.8% of total SNPs were located in the upstream regions, which the authors suspect is due to the definition of what constitutes as “upstream.” Missense variants are more frequent in the investigated casein region, with a proportion of 2.2% compared to the rest of the bovine genome (1.4%), which might point to more abundant genetic variation in the casein cluster compared to the whole genome. Overall, the casein cluster is very similar compared to the average cattle genome, with a few small deviations in the percentage of SNPs found in the upstream, missense, 3′-, and 5′-UTR as well as in splice sites.

In our analysis, we found 892 SNPs, of which 254 were present in DSN (28.4%). The allele frequencies across all SNPs clearly differentiate between the different cattle breeds. In upstream regulatory regions, no new variant was detected in DSN. Upstream variants in *CSN1S1*, *CSN2*, and *CSN3*, which might have regulatory effects on gene expression, have an allele frequency distribution in DSN similar to other breeds, and the allele frequencies of two variants in the upstream regions of *CSN1S2* are comparable to Danish Red. This is interesting because DSN and Danish Red have similar breeding goals towards a dual-purpose phenotype and the breeds show similar fat and protein percentages in milk. As such, it could be proposed that the similarities in the *CSN1S2* upstream regions could be influencing the expression level of *CSN1S2* in both breeds, leading to similarities in the protein composition of the milk from these breeds. The expression level of the *CSN1S2* gene variant of DSN/Danish Red should be further investigated in comparison to other breeds.

Six new DNA variants were detected in the intronic regions of *CSN1S1*, *CSN2*, and *CSN3*. Three out of these six new DNA variants were detected in two different *CSN2* intron regions in a single DSN bull only. Because of the relatively stringent quality filter for sequencing data of at least three reads to one allele, we are reasonably confident that these three SNPs are real. However, a sequencing failure in this animal cannot be fully excluded. Three additional new SNPs that were detected in DSN and other breeds are reliable because of their frequencies and their occurrence in different breeds.

### Casein Protein Variants

No variation was detected in the two α-caseins in DSN. In the 30 sequenced animals, only the CSN1S1*B and CSN1S2*A variants were detected, while in Holstein the protein variants CSN1S1*C and CSN1S2*D were detected at low frequencies. However, since the investigated DSN population was small, we cannot exclude additional α_S1_ and α_S2_ protein variants; for example, variant CSN1S1*C has recently been detected in DSN (Hohmann et al., 2018).

In other breeds selected for high milk yield, the CSN1S1*B variant was reported to be fixed (Caroli et al., 2003). For DSN, which is a dual-purpose breed, CSN1S1*B is the only variant detected in our study. Interestingly, Jersey cattle, which were selected for high fat and protein content, showed the lowest frequency for CSN1S1*B (51.9%) and the highest frequency for CSN1S1*C (44.8%), which might mean a positive effect on protein and fat content for the CSN1S1*C variant. Since CSN1S1*C was recently detected in DSN (Hohmann et al., 2018), this might provide an opportunity for DSN breeders to increase the percentage of milk fat and protein in DSN by actively searching for and breeding with animals carrying the CSN1S1*C variant.

The *A* *^1^* variant of the β-casein has a frequency of 82.9% in DSN, which is much higher than in other breeds. Compared to earlier results from the DSN population, an overestimation of this variant (DSN Brandenburg CSN2*A *^1^* = 67% frequency; Hohmann et al., 2018) could result from the small sample size in our data. This overestimation goes probably to the disadvantage of the β-casein variant *A* *^2^*, which we only detected by a frequency of 15.4% in DSN (DSN Brandenburg CSN2*A *^2^* = 31% frequency; Hohmann et al., 2018). The *I* variant of β-casein showed a frequency of 1.7% in our DSN population. While all casein variants that occur in DSN were also found in Holstein, the reverse situation is not true.

Since our study used SNPs to predict protein variants, we are not able to detect some known casein variants which can only be found using protein analysis. As an example, our study is unable to estimate the occurrence of CSN2*C since the dephosphorylation of Ser_35_P into a unphosphorylated Ser in *CSN2* happens posttranslational and can only be investigated at the protein molecule level (Gallinat et al., 2013). Other studies on the DSN population show the existence of the CSN2*B variant with low frequencies (DSN Brandenburg CSN2*B = 2% frequency; Hohmann et al., 2018). In further investigations, the sequence on protein level should be examined parallel to the DNA sequence.

With a frequency of 83.2%, the *A* variant of κ-casein is the most common in DSN, followed by CSN3*B (13.3%) and CSN3*E (3.5%). The variant frequencies agree with previous findings by Hohmann and colleagues (Hohmann et al., 2018). In contrast to Holstein, no additional κ-casein protein variant could be found in DSN. The *E* variant, which influences cheese making properties in a presumably negative way (Caroli et al., 2009), was detected in six breeds including DSN at a low frequency. A low frequency is also occurring in Holstein (4.6%) and Danish Red (3.6%). However, increasing the *E* variant in the population should be selected against in DSN.

### Casein Haplotype Frequencies in DSN Compared to Other Breeds

In DSN, *B-A* *^1^* *-A-A* is the most frequent casein haplotype with a frequency of 71.7%. This is due to the very high frequency of CSN2*A *^1^* (82.9%), which might be overestimated in our results. Studies with higher sample sizes showed similar results. Also, they detected the highest frequency (57%) for the shortened CSN1S1*B–CSN2*A *^1^*–CSN3*A haplotype in DSN (Hohmann et al., 2018). The 57% estimate should be considered the more reliable estimate as it is based on a larger sample size. The most common comprehensive casein haplotype in British Friesian was also *B-A* *^1^* *-A-A*, with a frequency of 60% (Jann et al., 2004), which is similar to the frequency found in DSN. In contrast to DSN, the protein variants CSN2*I and CSN3*E were not detected in British Frisian.

The haplotype *B-A* *^2^* *-A-A* is the most common in Holstein (53.1%) and several other *B. taurus* breeds (Limousin 41.9%, Angus 64.4%, Hereford 35.9%, Charolais 33.1%, Simmental 44.6%, Fleckvieh 41.5%, and Gelbvieh 49.0%), and the estimated frequencies of the casein protein variants reported in this paper are comparable to frequencies found in the literature, e.g., for Aberdeen Angus (51.1%) (Jann et al., 2004) or Italian Holsteins (CSN1S1*B-CSN2*A *^2^*-CSN3*A = 48%) (Boettcher et al., 2004). For Brown Swiss, the haplotype *B-A* *^2^* *-A-B* with a frequency of 50% is identical to results in the literature for the shortened haplotype CSN1S1*B-CSN2*A *^2^* -CSN3*B in Italian Brown Swiss (Boettcher et al., 2004). The cattle populations within the 1000 Bull Genomes Project seem to adequately represent the respective cattle breeds.

Further investigation should investigate the effect of different haplotypes in DSN on milk yield and protein and fat percentage. However, the current sample size would not lead to significant results. A previous investigation of casein variants with >600 DSN found no significant results based on the β- and κ-casein genotype (Freyer et al., 1999).

## Conclusion

Few of the already known casein protein variants, α_S1_ (*B*), β (*A* *^1^*, *A* *^2^*, and *I*), α_S2_ (*A*), and κ (*A*, *B*, and *E*), were detected in DSN using whole-genome sequencing data. This study is the first to find the CSN2*I variant in DSN. Besides the detection of this new variant, we confirm previous findings by Hohmann and colleagues that the most common casein cluster haplotype in DSN is *B-A* *^1^* *-A-A*. Based on the casein haplotype, DSN clusters together with Danish Red.

DSN cattle is remarkably different from the other investigated *B. taurus* breeds by having a high frequency of the CSN2*A *^1^* variant. The preferred protein variants CSN2*A *^2^* for potentially improving human health and CSN3*B for better cheese making properties were detected at low frequencies in the DSN breed. Our study found a large and untapped potential for DSN breeders to select and increase beneficial protein variants. However, selection for these variants could also (negatively) influence other important traits (e.g., protein and fat percentage or milk yield).

Because of its low variability, the α_S2_ protein is often omitted from casein studies. In our study of 14 breeds, we also come to the same conclusion that variability in α_S2_ is low and can be disregarded when investigating protein variants. However, we found a number of upstream genetic variations which show a similarity between the dual-purpose breeds DSN and Danish Red. These upstream variants might influence expression of the *CSN1S2* gene and should be investigated further.

## Data Availability Statement

All data required to reproduce the analysis, results, and conclusions can be requested from the authors at this point in time, since access to the 1000 Bull Genomes data is currently only available to partners. However, the 1000 Bull Genomes consortium will make the whole genome sequencing data available publicly when data collection and analysis is completed.

## Ethics Statement

Ethical review and approval was not required for the animal study because samples are collected based on routine procedures on these farm animals. Ear tags were taken as part of the required registration procedure, blood samples were taken by a trained veterinarian to perform standard health recording. Semen from bulls was acquired under routine conditions as part of the normal operation of RBB as an artificial insemination company.

## Author Contributions

SM, PK, DA, and GB designed the study. SM interpreted the data and drafted the manuscript. PK performed all computational and statistical analysis. DA, PK, and GB helped draft the manuscript. All authors read and approved the final manuscript.

## Funding

This project is funded by the German Federal Ministry of Food and Agriculture (BLE) and Federal Program of Ecological Agriculture (BÖLN) (funding number 2815NA010). We acknowledge support by the German Research Foundation (DFG) and the Open Access Publication Fund of Humboldt-Universität zu Berlin.

## Conflict of Interest

SM is an employee of the RBB Rinderproduktion Berlin-Brandenburg GmbH, a cattle breeders association which produces semen and provides services to DSN and Holstein cattle breeders in the Berlin/Brandenburg area.

The remaining authors declare that the research was conducted in the absence of any commercial or financial relationships that could be construed as a potential conflict of interest.
