# Supplementary material for: DNA Sequence Variants and Protein Haplotypes of Casein Genes in German Black Pied Cattle (DSN)
Source: Front Genet. 2019 Nov 8;10:1129. doi: 10.3389/fgene.2019.01129 (PMC6857469; doi:10.3389/fgene.2019.01129)

Supplementary Material

# Supplementary Files

Supplementary File 1. RScripts for the analysis and visualization of the data.

# Supplementary Tables

Supplementary Table 1. Genomic position of casein genes.

| Gene | Ensembl | Chr | Start | End | Strand |
| --- | --- | --- | --- | --- | --- |
| CSN1S1 | ENSBTAG00000007695 | 6 | 87141556 | 87159096 | + |
| CSN1S2 | ENSBTAG00000005005 | 6 | 87262457 | 87280936 | + |
| CSN2 | ENSBTAG00000002632 | 6 | 87188025 | 87179502 | - |
| CSN3 | ENSBTAG00000039787 | 6 | 87378398 | 87392750 | + |

Supplementary Table 2. Number of variants (proportion in parentheses) found in the casein genes *CSN1S1, CSN1S2, CSN2,* and *CSN3* and their 1000 bp upstream region.

| Variant type | *CSN1S1* | *CSN1S2* | *CSN2* | *CSN3* | Total |
| --- | --- | --- | --- | --- | --- |
| Upstream | 22 (6.9%) | 8 (4.2%) | 10 (7.2%) | 12 (4.9%) | 52 (5.8%) |
| 5’-UTR | 2 (0.6%) | 2 (1.1%) | 0 (0%) | 0 (0%) | 4 (0.4%) |
| Intron | 283 (88.2%) | 167 (88.4) | 116 (83.5%) | 213 (87.7%) | 779 (87.3%) |
| Missense | 3 (0.9%) | 3 (1.6%) | 7 (5.0%) | 7 (2.9%) | 20 (2.2%) |
| Synonymous | 3 (0.9%) | 4 (2.1%) | 3 (2.2%) | 1 (0.4%) | 11 (1.2%) |
| 3’-UTR | 5 (1.6%) | 3 (1.6%) | 3 (2.2%) | 9 (3.7%) | 20 (2.2%) |
| Splice region | 3 (0.9%) | 2 (1.1%) | 0 (0%) | 1 (0.4%) | 6 (0.7%) |
| Total | 321 (100%) | 189 (100%) | 139 (100%) | 243 (100%) | 892 (100%) |

**Supplementary Table 3.** Sequence variants in exon flanking regions of bovine CSN1S1, CSN2, CSN1S2, and CSN3 and their alternative allele frequency in DSN compared to HF and other breeds.
^a^ Positions of amino acids according to the reference protein sequence from Ensembl Release 93, positions in the mature protein are given in parentheses.
^b^ Bos Taurus autosome (BTA) CSN1S1*B (ENSBTAG00000007695), CSN2*A^2^ (ENSBTAG00000002632), CSN1S2*A (ENSBTAG00000005005), CSN3*A (ENSBTAG00000039787).

| Gene with  amino acid  variants^a^ | BTA  position^b^ | Reference  allele | Alternative  allele | Location and type of variant | SNP ID | Alternative allele frequency | | |
| --- | --- | --- | --- | --- | --- | --- | --- | --- |
|  |  |  |  |  |  | DSN | HF | all breeds |
| *CSN1S1*B*, | 6:87141369 | G | T | Upstream | rs458438593 | 0.017 | 0.006 | 0.004 |
| reference | 6:87141933 | T | G | Intron 1/18 | rs378000526 | 0.067 | 0.069 | 0.045 |
|  | 6:87142093 | T | C | Intron 1/18 | rs433672890 | 0.533 | 0.106 | 0.058 |
|  | 6:87142407 | C | T | Intron 1/18 | rs472722377 | 0.536 | 0.104 | 0.061 |
|  | 6:87142957 | T | C | Intron 1/18 | rs136894684 | 0.067 | 0.116 | 0.148 |
|  | 6:87143269 | T | C | Intron 2/18 | rs385123174 | 0.067 | 0.050 | 0.080 |
|  | 6:87143341 | A | T | Intron 2/18 | rs378098872 | 0.603 | 0.289 | 0.258 |
|  | 6:87143510 | T | C | Intron 2/18 | rs133920213 | 0.741 | 0.493 | 0.481 |
|  | 6:87144039 | G | T | Intron 2/18 | rs440780601 | 0.083 | 0.056 | 0.045 |
|  | 6:87144946 | C | T | Intron 2/18 | rs385533935 | 0.033 | 0.029 | 0.070 |
|  | 6:87145250 | T | C | Intron 3/18 | rs110899052 | 0.724 | 0.502 | 0.492 |
|  | 6:87145489 | A | G | Intron 3/18 | rs474566133 | 0.533 | 0.113 | 0.062 |
|  | 6:87146688 | T | A | Intron 5/18 | rs384783873 | 0.033 | 0.029 | 0.064 |
|  | 6:87146849 | C | T | Intron 5/18 | rs474629587 | 0.159 | 0.090 | 0.070 |
|  | 6:87146864 | G | T | Intron 5/18 | rs463486835 | 0.034 | 0.051 | 0.028 |
|  | 6:87146865 | A | T | Intron 5/18 | rs470921937 | 0.033 | 0.016 | 0.008 |
|  | 6:87147250 | G | A | Intron 6/18 | - | 0.033 | 0.001 | 0.001 |
|  | 6:87147499 | A | G | Intron 6/18 | rs384275664 | 0.067 | 0.064 | 0.035 |
|  | 6:87147828 | T | A | Intron 7/18 | rs210334109 | 0.067 | 0.062 | 0.041 |
|  | 6:87148121 | T | C | Intron 7/18 | rs447300666 | 0.533 | 0.107 | 0.061 |
|  | 6:87148327 | A | T | Intron 7/18 | rs109220469 | 0.650 | 0.359 | 0.311 |
|  | 6:87148883 | G | C | Intron 8/18 | rs209399459 | 0.067 | 0.064 | 0.048 |
|  | 6:87149208 | G | C | Intron 8/18 | rs437114477 | 0.052 | 0.063 | 0.046 |
|  | 6:87149241 | C | A | Intron 8/18 | rs457190750 | 0.067 | 0.065 | 0.035 |
|  | 6:87149357 | G | A | Intron 8/18 | rs109165367 | 0.067 | 0.104 | 0.146 |
|  | 6:87149389 | G | A | Intron 8/18 | rs383334452 | 0.067 | 0.064 | 0.045 |
|  | 6:87150259 | C | T | Intron 9/18 | rs110664549 | 0.100 | 0.234 | 0.208 |
|  | 6:87151533 | C | G | Intron 11/18 | rs210112991 | 0.768 | 0.546 | 0.585 |
|  | 6:87151585 | C | T | Intron 11/18 | rs209267342 | 0.052 | 0.061 | 0.043 |
|  | 6:87151935 | C | G | Intron 11/18 | rs381017130 | 0.052 | 0.064 | 0.042 |
|  | 6:87152046 | C | T | Intron 11/18 | rs457902649 | 0.036 | 0.069 | 0.034 |
|  | 6:87152150 | T | C | Intron 11/18 | rs516165119 | 0.050 | 0.001 | 0.001 |
|  | 6:87152621 | C | T | Intron 12/18 | rs110440863 | 0.724 | 0.499 | 0.489 |
|  | 6:87153292 | C | G | Intron 13/18 | rs460449688 | 0.536 | 0.105 | 0.058 |
|  | 6:87153613 | A | G | Intron 13/18 | rs465560348 | 0.533 | 0.109 | 0.061 |
|  | 6:87154270 | A | G | Intron 13/18 | rs438917711 | 0.534 | 0.108 | 0.060 |
|  | 6:87154277 | A | C | Intron 13/18 | rs378034503 | 0.033 | 0.033 | 0.073 |
|  | 6:87154387 | C | T | Intron 13/18 | rs137349211 | 0.067 | 0.112 | 0.147 |
|  | 6:87154501 | G | A | Intron 14/18 | rs382813379 | 0.067 | 0.065 | 0.044 |
|  | 6:87154594 | A | G | Intron 14/18 | rs109193501 | 0.050 | 0.131 | 0.245 |
|  | 6:87155332 | C | T | Splice Region, Intron 14/18 | - | 0.017 | 0.004 | 0.002 |
|  | 6:87155863 | C | G | Intron 15/18 | rs383429876 | 0.017 | 0.092 | 0.069 |
|  | 6:87156132 | C | G | Intron 15/18 | rs110258698 | 0.750 | 0.535 | 0.579 |
|  | 6:87156194 | A | T | Intron 15/18 | rs379518794 | 0.033 | 0.033 | 0.080 |
|  | 6:87156953 | C | T | Intron 16/18 | rs380635300 | 0.067 | 0.052 | 0.079 |
|  | 6:87157421 | A | G | Intron 17/18 | rs110608375 | 0.036 | 0.057 | 0.086 |
|  | 6:87158307 | G | A | Intron 18/18 | rs209385308 | 0.033 | 0.033 | 0.067 |
|  | 6:87158448 | C | T | Intron 18/18 | rs108969776 | 0.067 | 0.076 | 0.110 |
|  | 6:87159082 | A | G | Exon 19/19 3' UTR | rs211141048 | 0.033 | 0.032 | 0.074 |
| *CSN2*A^2^*,  reference | 6:87179786 | A | T | Exon 9/9 3' UTR | rs109439791 | 0.033 | 0.088 | 0.123 |
|  | 6:87179811 | C | T | Exon 9/9 3' UTR | rs110688365 | 0.033 | 0.088 | 0.122 |
|  | 6:87180394 | A | G | Intron 8/8 | rs379780371 | 0.033 | 0.089 | 0.121 |
|  | 6:87180474 | A | G | Intron 8/8 | rs110223653 | 0.817 | 0.472 | 0.463 |
|  | 6:87180731 | A | C | Intron 7/8 | rs110672723 | 0.783 | 0.354 | 0.306 |
|  | 6:87180975 | C | T | Intron 7/8 | rs110792714 | 0.033 | 0.087 | 0.119 |
|  | 6:87181031 | A | G | Intron 7/8 | rs110466181 | 0.833 | 0.552 | 0.516 |
|  | 6:87181090 | T | C | Intron 7/8 | rs381421757 | 0.017 | 0.062 | 0.038 |
| *CSN2*I* Met108Leu (Met93Leu) | 6:87181542 | T | G | Exon 7/9 non-synonymous | rs109299401 | 0.017 | 0.059 | 0.036 |
| *CSN2*A^1^* His82Pro  (His67Pro) | 6:87181619 | G | T | Exon 7/9 non-synonymous | rs43703011 | 0.827 | 0.340 | 0.296 |
|  | 6:87182259 | C | G | Intron 6/8 | rs110102622 | 0.033 | 0.041 | 0.067 |
|  | 6:87182740 | G | T | Intron 6/8 | rs110496027 | 0.033 | 0.092 | 0.120 |
|  | 6:87182857 | T | C | Intron 6/8 | rs109780890 | 0.783 | 0.354 | 0.308 |
|  | 6:87183113 | A | T | Intron 5/8 | rs110295588 | 0.817 | 0.471 | 0.459 |
|  | 6:87183306 | C | A | Intron 4/8 | rs109526437 | 0.845 | 0.558 | 0.572 |
|  | 6:87183612 | C | T | Intron 4/8 | rs110564693 | 0.793 | 0.350 | 0.306 |
|  | 6:87184038 | G | A | Intron 4/8 | rs380630763 | 0.033 | 0.088 | 0.113 |
|  | 6:87184066 | A | G | Intron 4/8 | rs383297660 | 0.833 | 0.549 | 0.512 |
|  | 6:87184080 | G | C | Intron 4/8 | rs378689750 | 0.857 | 0.556 | 0.569 |
|  | 6:87184171 | G | A | Intron 4/8 | rs81109614 | 0.852 | 0.472 | 0.462 |
|  | 6:87184548 | T | C | Intron 4/8 | rs81184820 | 0.793 | 0.350 | 0.307 |
|  | 6:87184676 | G | C | Intron 4/8 | rs109000439 | 0.033 | 0.300 | 0.085 |
|  | 6:87184912 | C | G | Intron 4/8 | - | 0.017 | 0.000 | 0.000 |
|  | 6:87185025 | T | A | Intron 4/8 | - | 0.017 | 0.000 | 0.000 |
|  | 6:87185894 | G | A | Intron 2/8 | rs110850982 | 0.793 | 0.317 | 0.199 |
|  | 6:87186177 | G | A | Intron 1/8 | - | 0.017 | 0.000 | 0.000 |
|  | 6:87186275 | A | G | Intron 1/8 | rs109804899 | 0.033 | 0.046 | 0.040 |
|  | 6:87186451 | G | A | Intron 1/8 | rs464982173 | 0.100 | 0.007 | 0.021 |
|  | 6:87186551 | G | A | Intron 1/8 | rs379595622 | 0.017 | 0.060 | 0.042 |
|  | 6:87186789 | T | A | Intron 1/8 | rs208169228 | 0.050 | 0.064 | 0.028 |
|  | 6:87187426 | C | A | Intron 1/8 | rs385114839 | 0.017 | 0.054 | 0.033 |
|  | 6:87187581 | T | C | Intron 1/8 | rs109091301 | 0.845 | 0.548 | 0.513 |
|  | 6:87188128 | G | C | Upstream | rs108993011 | 0.017 | 0.065 | 0.039 |
| *CSN1S2*A*, | 6:87262140 | G | A | Upstream | rs110158897 | 0.862 | 0.369 | 0.217 |
| reference | 6:87262450 | A | C | Upstream | rs136328312 | 0.117 | 0.472 | 0.516 |
|  | 6:87262558 | A | G | Intron 1/17 | rs386113802 | 0.017 | 0.063 | 0.037 |
|  | 6:87263065 | G | A | Intron 1/17 | rs207864947 | 0.067 | 0.312 | 0.268 |
|  | 6:87263159 | T | C | Intron 1/17 | rs385035415 | 0.033 | 0.046 | 0.038 |
|  | 6:87263255 | G | C | Intron 1/17 | rs380536159 | 0.017 | 0.049 | 0.032 |
|  | 6:87263402 | C | T | Intron 1/17 | rs109606708 | 0.033 | 0.123 | 0.179 |
|  | 6:87263933 | G | A | Intron 1/17 | rs109269946 | 0.150 | 0.632 | 0.773 |
|  | 6:87264294 | T | C | Exon 2/18 5' UTR | rs109900747 | 0.117 | 0.471 | 0.559 |
|  | 6:87264624 | G | A | Intron 2/17 | rs109950192 | 0.033 | 0.045 | 0.041 |
|  | 6:87265002 | A | G | Intron 2/17 | rs209372951 | 0.207 | 0.658 | 0.797 |
|  | 6:87265427 | C | T | Intron 2/17 | rs207688167 | 0.103 | 0.478 | 0.509 |
|  | 6:87265836 | C | T | Intron 2/17 | rs110628707 | 0.207 | 0.657 | 0.796 |
|  | 6:87265871 | G | A | Intron 2/17 | rs471580574 | 0.033 | 0.015 | 0.006 |
|  | 6:87266390 | T | C | Intron 3/17 | rs480491637 | 0.067 | 0.007 | 0.006 |
|  | 6:87266799 | G | C | Intron 3/17 | rs379531807 | 0.033 | 0.051 | 0.058 |
|  | 6:87266870 | G | T | Intron 3/17 | rs133118527 | 0.117 | 0.469 | 0.518 |
|  | 6:87267144 | C | T | Intron 5/17 | rs110808655 | 0.150 | 0.631 | 0.775 |
|  | 6:87269654 | G | A | Intron 8/17 | rs383622192 | 0.033 | 0.045 | 0.037 |
|  | 6:87270082 | A | G | Intron 10/17 | rs110162599 | 0.967 | 0.923 | 0.947 |
|  | 6:87271085 | T | G | Intron 12/17 | rs109565340 | 0.125 | 0.630 | 0.776 |
|  | 6:87271686 | C | T | Intron 12/17 | rs136532031 | 0.033 | 0.115 | 0.166 |
|  | 6:87271868 | G | A | Intron 12/17 | rs381363035 | 0.067 | 0.313 | 0.264 |
|  | 6:87272033 | C | T | Intron 12/17 | rs110122319 | 0.150 | 0.631 | 0.774 |
|  | 6:87272114 | C | T | Intron 12/17 | rs133836703 | 0.103 | 0.471 | 0.516 |
|  | 6:87272568 | G | T | Intron 13/17 | rs109493928 | 0.150 | 0.627 | 0.776 |
|  | 6:87273754 | T | C | Intron 13/17 | rs432256660 | 0.050 | 0.019 | 0.011 |
|  | 6:87276023 | C | T | Intron 16/17 | rs109185641 | 0.150 | 0.630 | 0.773 |
|  | 6:87276027 | G | T | Intron 16/17 | rs380274739 | 0.033 | 0.050 | 0.055 |
|  | 6:87276866 | T | C | Intron 17/17 | rs109730929 | 0.150 | 0.625 | 0.738 |
|  | 6:87278256 | T | G | Intron 17/17 | rs801217221 | 0.017 | 0.062 | 0.020 |
|  | 6:87278260 | G | A | Intron 17/17 | rs456832476 | 0.052 | 0.020 | 0.011 |
|  | 6:87278394 | G | A | Intron 17/17 | rs381462400 | 0.100 | 0.319 | 0.303 |
|  | 6:87279173 | C | T | Intron 17/17 | rs381228720 | 0.850 | 0.365 | 0.249 |
|  | 6:87279375 | T | A | Intron 17/17 | rs209446283 | 0.138 | 0.630 | 0.740 |
|  | 6:87279994 | A | G | Intron 17/17 | rs133459561 | 0.033 | 0.118 | 0.178 |
|  | 6:87280088 | G | A | Intron 17/17 | rs109500451 | 0.150 | 0.624 | 0.736 |
|  | 6:87280346 | T | G | Intron 17/17 | rs134043709 | 0.103 | 0.471 | 0.518 |
|  | 6:87280796 | T | C | Exon 18/18 3' UTR | rs109261203 | 0.150 | 0.634 | 0.780 |
|  | 6:87280919 | G | A | Exon 18/18 3' UTR | rs109274107 | 0.150 | 0.623 | 0.734 |
| *CSN3*A*, | 6:87377971 | T | C | Upstream | rs109921787 | 0.103 | 0.108 | 0.217 |
| reference | 6:87378013 | T | C | Upstream | rs111012468 | 0.117 | 0.106 | 0.221 |
|  | 6:87378597 | G | C | Intron 1/4 | rs383784450 | 0.117 | 0.111 | 0.219 |
|  | 6:87378732 | A | G | Intron 1/4 | rs133770245 | 0.117 | 0.103 | 0.203 |
|  | 6:87378930 | C | T | Intron 1/4 | rs384519400 | 0.050 | 0.024 | 0.041 |
|  | 6:87379100 | G | A | Intron 1/4 | rs208444965 | 0.050 | 0.071 | 0.121 |
|  | 6:87379195 | C | A | Intron 1/4 | rs137468837 | 0.117 | 0.112 | 0.223 |
|  | 6:87379296 | A | G | Intron 1/4 | rs136258508 | 0.117 | 0.108 | 0.221 |
|  | 6:87379726 | C | T | Intron 1/4 | rs133814568 | 0.103 | 0.103 | 0.218 |
|  | 6:87379742 | A | G | Intron 1/4 | rs135678999 | 0.103 | 0.103 | 0.218 |
|  | 6:87379764 | T | G | Intron 1/4 | rs132910489 | 0.117 | 0.101 | 0.211 |
|  | 6:87379776 | G | A | Intron 1/4 | rs110923613 | 0.867 | 0.790 | 0.657 |
|  | 6:87379841 | C | A | Intron 1/4 | rs380838280 | 0.117 | 0.100 | 0.216 |
|  | 6:87379923 | C | T | Intron 1/4 | rs378592289 | 0.117 | 0.104 | 0.218 |
|  | 6:87379932 | G | T | Intron 1/4 | rs135413628 | 0.117 | 0.102 | 0.217 |
|  | 6:87380036 | C | T | Intron 1/4 | rs136574147 | 0.089 | 0.103 | 0.216 |
|  | 6:87380061 | C | T | Intron 1/4 | rs134181255 | 0.103 | 0.106 | 0.218 |
|  | 6:87380071 | C | G | Intron 1/4 | rs137625018 | 0.103 | 0.107 | 0.218 |
|  | 6:87380231 | C | T | Intron 1/4 | rs133437757 | 0.117 | 0.108 | 0.222 |
|  | 6:87380244 | G | A | Intron 1/4 | rs134028657 | 0.117 | 0.102 | 0.220 |
|  | 6:87380313 | C | T | Intron 1/4 | rs135217876 | 0.117 | 0.099 | 0.212 |
|  | 6:87380361 | G | A | Intron 1/4 | rs133275355 | 0.117 | 0.108 | 0.217 |
|  | 6:87380594 | G | T | Intron 1/4 | rs137310313 | 0.117 | 0.098 | 0.213 |
|  | 6:87380601 | G | A | Intron 1/4 | rs136263688 | 0.117 | 0.090 | 0.210 |
|  | 6:87380633 | A | C | Intron 1/4 | rs136730098 | 0.117 | 0.102 | 0.211 |
|  | 6:87380644 | T | C | Intron 1/4 | rs134611229 | 0.117 | 0.106 | 0.205 |
|  | 6:87380649 | C | T | Intron 1/4 | rs136474661 | 0.117 | 0.106 | 0.210 |
|  | 6:87380674 | A | C | Intron 1/4 | rs134231196 | 0.117 | 0.105 | 0.208 |
|  | 6:87380753 | A | C | Intron 1/4 | rs135489914 | 0.103 | 0.098 | 0.206 |
|  | 6:87380783 | T | A | Intron 1/4 | rs136419748 | 0.103 | 0.109 | 0.212 |
|  | 6:87380811 | T | G | Intron 1/4 | rs133764266 | 0.107 | 0.106 | 0.210 |
|  | 6:87380816 | C | T | Intron 1/4 | rs137466447 | 0.107 | 0.106 | 0.212 |
|  | 6:87380982 | A | T | Intron 2/4 | rs209219846 | 0.750 | 0.661 | 0.382 |
|  | 6:87380989 | A | T | Intron 2/4 | rs377836309 | 0.917 | 0.819 | 0.773 |
|  | 6:87381018 | A | G | Intron 2/4 | rs446176363 | 0.050 | 0.064 | 0.044 |
|  | 6:87381057 | A | G | Intron 2/4 | rs133071784 | 0.967 | 0.955 | 0.943 |
|  | 6:87381125 | C | T | Intron 2/4 | rs110486234 | 0.867 | 0.798 | 0.657 |
|  | 6:87381159 | G | A | Intron 2/4 | rs208952534 | 0.750 | 0.667 | 0.392 |
|  | 6:87381233 | T | C | Intron 2/4 | rs136886157 | 0.117 | 0.103 | 0.209 |
|  | 6:87381284 | G | C | Intron 2/4 | rs135031621 | 0.117 | 0.101 | 0.210 |
|  | 6:87381368 | A | G | Intron 2/4 | rs132864037 | 0.117 | 0.106 | 0.213 |
|  | 6:87381781 | G | A | Intron 2/4 | rs135696763 | 0.103 | 0.102 | 0.214 |
|  | 6:87382140 | T | C | Intron 2/4 | - | 0.283 | 0.046 | 0.055 |
|  | 6:87382171 | A | T | Intron 2/4 | rs132703805 | 0.103 | 0.105 | 0.206 |
|  | 6:87382339 | T | C | Intron 2/4 | rs461450736 | 0.121 | 0.091 | 0.190 |
|  | 6:87382358 | A | T | Intron 2/4 | rs382037958 | 0.103 | 0.088 | 0.184 |
|  | 6:87382446 | G | A | Intron 2/4 | rs385854541 | 0.117 | 0.098 | 0.204 |
|  | 6:87382546 | G | A | Intron 2/4 | rs380322207 | 0.117 | 0.108 | 0.204 |
|  | 6:87382617 | A | T | Intron 2/4 | rs382710835 | 1.000 | 1.000 | 1.000 |
|  | 6:87382646 | T | G | Intron 2/4 | rs385986804 | 1.000 | 1.000 | 1.000 |
|  | 6:87382649 | A | G | Intron 2/4 | rs380669780 | 0.103 | 0.108 | 0.208 |
|  | 6:87382865 | G | T | Intron 2/4 | rs110837855 | 0.750 | 0.667 | 0.400 |
|  | 6:87383137 | G | A | Intron 2/4 | rs133439202 | 0.133 | 0.104 | 0.212 |
|  | 6:87383339 | A | G | Intron 2/4 | rs136529315 | 0.103 | 0.099 | 0.200 |
|  | 6:87383398 | G | T | Intron 2/4 | rs136890625 | 0.117 | 0.104 | 0.207 |
|  | 6:87383506 | T | A | Intron 2/4 | rs381954874 | 0.103 | 0.102 | 0.214 |
|  | 6:87383906 | G | A | Intron 2/4 | rs137775658 | 0.117 | 0.104 | 0.210 |
|  | 6:87383990 | C | T | Intron 2/4 | rs133893740 | 0.117 | 0.097 | 0.211 |
|  | 6:87384151 | T | A | Intron 2/4 | rs135321568 | 0.117 | 0.099 | 0.206 |
|  | 6:87384158 | C | T | Intron 2/4 | rs132729880 | 1.000 | 0.974 | 0.972 |
|  | 6:87384419 | A | G | Intron 2/4 | rs133692042 | 0.103 | 0.106 | 0.218 |
|  | 6:87384587 | G | A | Intron 2/4 | rs384126063 | 0.768 | 0.662 | 0.377 |
|  | 6:87384676 | G | A | Intron 2/4 | rs474584611 | 0.033 | 0.000 | 0.010 |
|  | 6:87384917 | C | T | Intron 2/4 | rs135198477 | 0.117 | 0.065 | 0.198 |
|  | 6:87385199 | A | G | Intron 2/4 | rs109568664 | 0.232 | 0.337 | 0.588 |
|  | 6:87385233 | G | A | Intron 2/4 | rs110516603 | 0.241 | 0.335 | 0.597 |
|  | 6:87385355 | C | G | Intron 2/4 | rs132978733 | 0.117 | 0.106 | 0.211 |
|  | 6:87385474 | G | T | Intron 2/4 | rs109828676 | 0.750 | 0.663 | 0.408 |
|  | 6:87385492 | C | A | Intron 2/4 | rs210668711 | 0.750 | 0.657 | 0.404 |
|  | 6:87385639 | T | A | Intron 2/4 | rs109819107 | 0.750 | 0.657 | 0.398 |
|  | 6:87385989 | A | C | Intron 2/4 | rs110079521 | 0.778 | 0.665 | 0.383 |
|  | 6:87386044 | C | T | Intron 2/4 | rs135605515 | 0.759 | 0.668 | 0.406 |
|  | 6:87386081 | T | C | Intron 2/4 | rs382037034 | 0.103 | 0.102 | 0.205 |
|  | 6:87386110 | T | A | Intron 2/4 | rs383598174 | 0.100 | 0.145 | 0.259 |
|  | 6:87386111 | A | T | Intron 2/4 | rs377869400 | 0.117 | 0.102 | 0.206 |
|  | 6:87386169 | A | G | Intron 2/4 | rs136373661 | 0.034 | 0.018 | 0.009 |
|  | 6:87386170 | A | G | Intron 2/4 | rs459618023 | 0.688 | 0.566 | 0.343 |
|  | 6:87386448 | A | G | Intron 2/4 | rs211213909 | 0.750 | 0.668 | 0.400 |
|  | 6:87386469 | G | A | Intron 2/4 | rs133261182 | 0.117 | 0.095 | 0.204 |
|  | 6:87386490 | A | G | Intron 2/4 | rs135106622 | 0.862 | 0.783 | 0.649 |
|  | 6:87386517 | C | T | Intron 2/4 | rs136314233 | 0.103 | 0.096 | 0.194 |
|  | 6:87386635 | C | G | Intron 2/4 | rs383084285 | 0.778 | 0.656 | 0.401 |
|  | 6:87386752 | G | C | Intron 2/4 | rs109706526 | 0.750 | 0.664 | 0.412 |
|  | 6:87387054 | T | C | Intron 3/4 | rs110493160 | 0.879 | 0.793 | 0.658 |
|  | 6:87387077 | T | A | Intron 3/4 | rs137201578 | 0.103 | 0.107 | 0.213 |
|  | 6:87387208 | T | C | Intron 3/4 | rs110888103 | 0.867 | 0.802 | 0.659 |
|  | 6:87387418 | T | C | Intron 3/4 | rs210486066 | 0.759 | 0.667 | 0.401 |
|  | 6:87387537 | A | G | Intron 3/4 | rs211398682 | 0.867 | 0.802 | 0.654 |
|  | 6:87387585 | C | T | Intron 3/4 | rs208310470 | 0.867 | 0.785 | 0.649 |
|  | 6:87387721 | G | A | Intron 3/4 | rs134745241 | 0.103 | 0.089 | 0.183 |
|  | 6:87387791 | T | C | Intron 3/4 | rs110860864 | 0.768 | 0.692 | 0.390 |
|  | 6:87387869 | C | T | Intron 3/4 | rs382107247 | 0.759 | 0.682 | 0.367 |
|  | 6:87387870 | A | G | Intron 3/4 | rs382652853 | 0.759 | 0.687 | 0.393 |
|  | 6:87387877 | C | T | Intron 3/4 | rs135071629 | 0.103 | 0.078 | 0.199 |
|  | 6:87387947 | C | A | Intron 3/4 | rs381081726 | 0.867 | 0.802 | 0.662 |
|  | 6:87387979 | A | C | Intron 3/4 | rs209504329 | 0.867 | 0.789 | 0.650 |
|  | 6:87388033 | T | C | Intron 3/4 | rs455917561 | 0.050 | 0.054 | 0.037 |
|  | 6:87388064 | A | G | Intron 3/4 | rs379473589 | 0.750 | 0.657 | 0.374 |
|  | 6:87388076 | C | T | Intron 3/4 | rs383173653 | 0.750 | 0.641 | 0.373 |
|  | 6:87388976 | T | A | Intron 3/4 | rs471895482 | 0.054 | 0.019 | 0.014 |
|  | 6:87388981 | A | G | Intron 3/4 | rs521405133 | 0.033 | 0.058 | 0.063 |
|  | 6:87388982 | T | A | Intron 3/4 | rs797410810 | 0.107 | 0.032 | 0.025 |
|  | 6:87388998 | A | G | Intron 3/4 | rs1114935235 | 0.017 | 0.025 | 0.016 |
|  | 6:87389002 | G | A | Intron 3/4 | rs474240401 | 0.283 | 0.090 | 0.089 |
|  | 6:87389872 | T | C | Intron 3/4 | rs377840250 | 0.103 | 0.094 | 0.150 |
|  | 6:87389930 | G | A | Intron 3/4 | rs211550565 | 0.750 | 0.667 | 0.407 |
| *CSN3*B* Thr157Ile  (Thr136Ile) | 6:87390576 | T | C | Exon 4/5 non-synonymous | rs43703015 | 0.867 | 0.797 | 0.659 |
| Asp169Ala (Asp148Ala) | 6:87390612 | C | A | Exon 4/5 non-synonymous | rs43703016 | 0.867 | 0.807 | 0.665 |
| *CSN3*E* Ser176Gly (Ser155Gly) | 6:87390632 | A | G | Exon 4/5 non-synonymous | rs43703017 | 0.034 | 0.045 | 0.031 |
| *CSN3*A* Ala189Ala (Ala168Ala) | 6:87390673 | G | A | Exon 4/5 synonymous | rs110014544 | 0.867 | 0.795 | 0.664 |
|  | 6:87390681 | T | A | Exon 4/5 3' UTR | rs109787476 | 0.867 | 0.796 | 0.661 |
|  | 6:87390993 | C | T | Intron 4/4 | rs110716489 | 0.750 | 0.661 | 0.404 |
|  | 6:87391018 | A | G | Intron 4/4 | rs109636009 | 0.867 | 0.789 | 0.658 |
|  | 6:87391201 | T | C | Intron 4/4 | rs110975631 | 0.867 | 0.788 | 0.656 |
|  | 6:87391208 | A | C | Intron 4/4 | rs110314968 | 0.867 | 0.788 | 0.654 |
|  | 6:87391483 | G | T | Intron 4/4 | rs109663346 | 0.867 | 0.782 | 0.654 |
|  | 6:87391584 | G | A | Intron 4/4 | rs134914762 | 0.117 | 0.101 | 0.216 |
|  | 6:87391701 | A | G | Intron 4/4 | rs109190605 | 0.750 | 0.657 | 0.402 |
|  | 6:87391848 | A | G | Intron 4/4 | rs109122729 | 0.867 | 0.787 | 0.655 |
|  | 6:87391903 | G | A | Intron 4/4 | rs109311071 | 0.867 | 0.794 | 0.658 |
|  | 6:87392123 | T | C | Intron 4/4 | rs207657048 | 0.750 | 0.665 | 0.400 |
|  | 6:87392211 | A | T | Intron 4/4 | rs136944141 | 0.867 | 0.781 | 0.648 |
|  | 6:87392343 | G | C | Intron 4/4 | rs109344408 | 0.867 | 0.794 | 0.657 |
|  | 6:87392391 | T | G | Intron 4/4 | rs135227383 | 0.103 | 0.104 | 0.213 |
|  | 6:87392502 | G | A | Intron 4/4 | rs208419263 | 0.867 | 0.795 | 0.661 |
|  | 6:87392580 | T | C | Exon 5/5 Splice Region, 3' UTR | rs209024052 | 0.867 | 0.799 | 0.661 |
|  | 6:87392591 | T | C | Exon 5/5 3' UTR | rs209359743 | 0.867 | 0.801 | 0.662 |
|  | 6:87392592 | G | A | Exon 5/5 3' UTR | rs135528518 | 0.867 | 0.803 | 0.662 |
|  | 6:87392635 | G | A | Exon 5/5 3' UTR | rs135796226 | 0.133 | 0.204 | 0.338 |
|  | 6:87392665 | T | C | Exon 5/5 3' UTR | rs134221650 | 0.241 | 0.338 | 0.598 |
|  | 6:87392674 | C | T | Exon 5/5 3' UTR | rs136843602 | 0.133 | 0.208 | 0.339 |
|  | 6:87392713 | A | G | Exon 5/5 3' UTR | rs134516686 | 0.133 | 0.214 | 0.340 |

Supplementary Table 4. Frequencies of protein variants in α_S1_ and α_S2_ per breed.

| N individuals | | **α_S1_** | | | **α_S2_** | |
| --- | --- | --- | --- | --- | --- | --- |
|  |  | B | C | other | A | other |
| DSN | 30 | 1.000 |  |  | 1.000 |  |
| Holstein | 541 | 0.995 | 0.005 |  | 0.997 | 0.003 |
| Danish Red | 56 | 0.991 | 0.009 |  | 0.946 | 0.054 |
| Jersey | 66 | 0.519 | 0.448 | 0.033 | 1.000 |  |
| Gelbvieh | 52 | 1.000 |  |  | 0.878 | 0.122 |
| Brown Swiss | 148 | 0.881 | 0.119 |  | 0.952 | 0.048 |
| Montbéliarde | 54 | 0.971 | 0.029 |  | 1.000 |  |
| Normande | 44 | 0.720 | 0.256 | 0.024 | 1.000 |  |
| Fleckvieh | 53 | 0.885 | 0.115 |  | 1.000 |  |
| Simmental | 217 | 0.917 | 0.083 |  | 0.998 | 0.002 |
| Charolais | 127 | 0.939 | 0.061 |  | 0.988 | 0.012 |
| Hereford | 75 | 0.972 | 0.028 |  | 1.000 |  |
| Angus | 276 | 0.946 | 0.052 | 0.002 | 0.925 | 0.075 |
| Limousin | 82 | 0.877 | 0.104 | 0.019 | 0.968 | 0.032 |
| Total | 1821 | 0.933 | 0.064 | 0.003 | 0.976 | 0.024 |

Supplementary Table 5. Frequencies of protein variants in β casein per breed.

| Breed | N individuals | A1 | A2 | B | I | F | Other |
| --- | --- | --- | --- | --- | --- | --- | --- |
| DSN | 30 | 0.829 | 0.154 |  | 0.017 |  |  |
| Holstein | 541 | 0.303 | 0.596 | 0.028 | 0.058 | 0.003 | 0.012 |
| Danish Red | 56 | 0.457 | 0.388 | 0.019 | 0.000 | 0.136 |  |
| Jersey | 66 | 0.114 | 0.611 | 0.227 | 0.049 |  |  |
| Gelbvieh | 52 | 0.188 | 0.761 | 0.011 | 0.014 | 0.010 | 0.017 |
| Brown Swiss | 148 | 0.093 | 0.745 | 0.162 | 0.000 |  |  |
| Montbéliarde | 54 | 0.070 | 0.412 | 0.355 | 0.152 |  | 0.011 |
| Normande | 44 | 0.099 | 0.471 | 0.232 | 0.154 |  | 0.044 |
| Fleckvieh | 53 | 0.286 | 0.638 | 0.021 | 0.031 |  | 0.023 |
| Simmental | 217 | 0.212 | 0.710 | 0.044 | 0.019 |  | 0.015 |
| Charolais | 127 | 0.202 | 0.680 | 0.080 | 0.009 |  | 0.029 |
| Hereford | 75 | 0.338 | 0.417 | 0.239 | 0.007 |  |  |
| Angus | 276 | 0.042 | 0.947 | 0.002 | 0.009 |  |  |
| Limousin | 82 | 0.165 | 0.748 | 0.036 | 0.021 |  | 0.030 |
| Total | 1821 | 0.214 | 0.668 | 0.068 | 0.035 | 0.005 | 0.008 |

**Supplementary Table 6.** Frequencies of protein variants in κ casein per breed.

| Breed | N individuals | A | B | E | Other |
| --- | --- | --- | --- | --- | --- |
| DSN | 30 | 0.832 | 0.133 | 0.035 |  |
| Holstein | 541 | 0.743 | 0.209 | 0.046 | 0.002 |
| Danish Red | 56 | 0.726 | 0.238 | 0.036 |  |
| Jersey | 66 | 0.040 | 0.960 |  |  |
| Gelbvieh | 52 | 0.599 | 0.390 |  | 0.011 |
| Brown Swiss | 148 | 0.279 | 0.674 |  | 0.047 |
| Montbéliarde | 54 | 0.510 | 0.490 |  |  |
| Normande | 44 | 0.154 | 0.846 |  |  |
| Fleckvieh | 53 | 0.844 | 0.145 |  | 0.011 |
| Simmental | 217 | 0.673 | 0.308 | 0.005 | 0.014 |
| Charolais | 127 | 0.446 | 0.510 |  | 0.045 |
| Hereford | 75 | 0.696 | 0.304 |  |  |
| Angus | 276 | 0.770 | 0.121 | 0.103 | 0.006 |
| Limousin | 82 | 0.476 | 0.449 | 0.007 | 0.068 |
| Total | 1821 | 0.619 | 0.336 | 0.031 | 0.013 |

**Supplementary Table 7** – Comparison of the casein cluster SNP annotation to the whole 1000 Bull Genomes data (Hayes and Daetwyler, 2019)

| **Annotation** | **SNP** | **[%] incl. Intergenic** | **[%] excl. Intergenic** | **Casein region** |
| --- | --- | --- | --- | --- |
| intergenic_variant | 28,353,891 | 66.1 | - |  |
| **intron_variant** | **11,232,495** | **26.2** | **84.7** | **87.3** |
| **upstream_gene_variant** | **1,510,605** | **3.5** | **11.4** | **5.8** |
| downstream_gene_variant | 1,282,230 | 3.0 |  |  |
| **missense_variant** | **185,046** | **0.4** | **1.4** | **2.2** |
| **synonymous_variant** | **179,442** | **0.4** | **1.4** | **1.2** |
| **3_prime_UTR_variant** | **99,283** | **0.2** | **0.7** | **2.2** |
| frameshift_variant |  | 0.0 | 0.0 |  |
| inframe_deletion |  | 0.0 | 0.0 |  |
| **splice_region_variant** | **32,194** | **0.1** | **0.2** | **0.7** |
| **5_prime_UTR_variant** | **23,431** | **0.1** | **0.2** | **0.4** |
| non_coding_transcript_exon_variant | 12,878 | 0.0 | 0.1 |  |
| inframe_insertion |  | 0.0 | 0.0 |  |
| stop_gained | 3,831 | 0.0 | 0.0 |  |
| splice_donor_variant | 1,876 | 0.0 | 0.0 |  |
| splice_acceptor_variant | 1,618 | 0.0 | 0.0 |  |
| mature_miRNA_variant | 407 | 0.0 | 0.0 |  |
| start_lost | 292 | 0.0 | 0.0 |  |
| stop_lost | 257 | 0.0 | 0.0 |  |
| coding_sequence_variant | 243 | 0.0 | 0.0 |  |
| stop_retained_variant | 126 | 0.0 | 0.0 |  |
| non_coding_transcript_variant | 82 | 0.0 | 0.0 |  |
| **Total** | **42,920,227** |  |  |  |

# Supplementary Figures

**Supplementary Figure 1 –** Pie chart comparison between 1000 bulls and the casein cluster

**Supplementary figure 2** – Dendrogram based on whole genome data


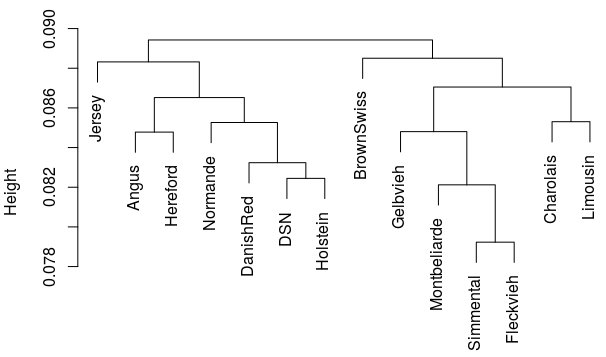

Supplement: Supplementary file 2 [file Table_1.docx]
